# Clinical Efficacy and Safety of Fosmanogepix in Patients with Invasive and Refractory Fungal Infections: A Systematic Review

**DOI:** 10.3390/jof12100733

**Published:** 2026-10-01

**Authors:** Felix Bratosin, Iulia Bogdan, Silvius Alexandru Pescariu

**Affiliations:** 1 Transplant Infectious Diseases, Division of Infectious Diseases, McGovern Medical School at UTHealth Houston, Houston, TX 77030, USA; felix.bratosin@uth.tmc.edu; 2 Department XIII, Discipline of Infectious Diseases, “Victor Babes” University of Medicine and Pharmacy Timisoara, 2 Eftimie Murgu Square, 300041 Timisoara, Romania; 3 Cardiology II, Department VI—Cardiology, “Victor Babes” University of Medicine and Pharmacy Timisoara, 2 Eftimie Murgu Square, 300041 Timisoara, Romania; pescariu.alexandru@umft.ro

**Keywords:** fosmanogepix, antifungal agents, invasive fungal infections, fusariosis, candidemia, aspergillosis

## Abstract

Background: Fosmanogepix is a first-in-class Gwt1 inhibitor with oral and intravenous formulations and broad in vitro activity, but its clinical evidence is scattered across small trials and case reports. We reviewed all identifiable human data from the searched sources on its efficacy and safety in serious fungal infections. Methods: PubMed, Google Scholar, ClinicalTrials.gov, congress supplements, and citation lists were searched for publications available up to 1 September 2026. Any report of fosmanogepix given for a fungal infection with a reported outcome was eligible. Quality was appraised with JBI checklists, findings were synthesised narratively and stratified by study design, certainty was rated for each indication with a GRADE-informed approach, and scenario analyses explored patient duplication and reporting bias. Results: Thirty studies (34 reports) describing approximately 223 reported patient-episodes (an estimated 171 to 214 unique patients under duplication scenarios) were included: three phase 2 trials (n = 51), seven expanded-access or cohort series (n = 151) and 20 case-level reports (n = 21). Adjudicated treatment success was 16/20 (80%) in candidaemia and 8/9 (89%) in *Candida auris* candidaemia; in refractory invasive mould disease, global response was 8/20 (40%) with 25% Day-42 mortality. Physician-assessed favourable responses were 13/17 (76%) and 4/7 (57%) in aspergillosis, 38/50 (76%) and 26/32 (81%) in fusariosis, 7/11 (64%) in mucormycosis, and 8/11 (73%) in coccidioidomycosis. Of 21 individually described patients, 17 were alive at last report. Gastrointestinal intolerance was the main adverse effect, with drug-related discontinuation in 3/21 mould-disease trial participants. GRADE-informed certainty was low for candidaemia other than *C. auris* candidaemia and for refractory mould disease, and very low for every other indication. Conclusions: Uncontrolled data provide signals of activity, not evidence of comparative effectiveness, in candidaemia, including *C. auris*, and in fusariosis and other resistant mould infections, with acceptable tolerability during prolonged use. Small samples, absent comparators, overlapping cohorts, and probable publication bias limit the evidence.

## 1. Introduction

Invasive fungal diseases remain a leading cause of death in patients with haematological malignancies, transplant recipients, and critically ill patients, and increasingly affect healthy people exposed to contaminated procedures or trauma. The World Health Organization fungal priority pathogens list highlights the shortage of effective drugs, the emergence of resistance and the toxicity of existing agents as reasons for urgent action [1]. Only three systemic antifungal classes support routine treatment, each with recognised limitations [2]. Intrinsically multidrug-resistant moulds such as *Fusarium solani* species complex and *Lomentospora prolificans* are treated with combination regimens of uncertain benefit, and mortality in disseminated disease has exceeded 60% [3,4].

Fosmanogepix is the prodrug of manogepix, an inhibitor of the fungal Gwt1 enzyme in glycosylphosphatidylinositol biosynthesis, a target absent from human cells [5,6]. Manogepix shows low inhibitory concentrations against most *Candida* spp., *Cryptococcus* spp., *Aspergillus* spp. including azole-resistant strains, *Fusarium* spp., *Scedosporium* spp. and *L. prolificans*, with variable activity against Mucorales, and it retains activity against pan-resistant *Candida auris* [7,8,9,10,11,12]. The prodrug has high oral bioavailability, a long half-life and wide tissue distribution [13,14,15]. In animal models, it was effective in candidiasis, aspergillosis, scedosporiosis, fusariosis, mucormycosis, and ocular or central nervous system infection [16,17,18,19,20,21], and combination with liposomal amphotericin B improved outcomes in experimental mould infection [22]. Phase 1 studies defined the loading and maintenance regimens and a modest interaction profile [23], and a phase 1b study confirmed tolerability in neutropenic leukaemia patients without evaluating treatment of infection [24]. Phase 3 trials in candidaemia and invasive mould infection are ongoing [25,26].

Clinical evidence comes from three single-arm phase 2 trials in candidaemia, *C. auris* candidaemia and invasive mould disease, each with between nine and 21 participants [27,28,29]. An expanded-access programme has supplied the drug to many more patients with no other treatment options, and this experience has been reported piecemeal as congress abstracts, an outbreak investigation of iatrogenic *Fusarium* meningitis, a coccidioidomycosis series and single-patient reports [30,31,32,33]. The reports raise unresolved questions. The 40% adjudicated response and 33% discontinuation rate in the mould trial contrast with physician-reported responses above 70% in expanded access [29,34]. Fatal progression of *L. prolificans* infection despite a very low manogepix concentration questioned whether in vitro susceptibility predicts response in deep-seated disease [35], and gastrointestinal intolerance requiring dose splitting recurs in the case literature [36,37].

No systematic review has assembled and appraised these human data. The two indexed systematic reviews that mention the drug address in vitro and animal data for *C. auris* and *Fusarium* spp. [38,39], the 2020 review labelled as systematic predated any efficacy trial [5], and recent narrative reviews used no reproducible search, omitted the abstracts and outbreak reports that contain most reported patient-episodes, and did not appraise quality [2,40,41]. Clinicians considering expanded access for a patient with a pan-resistant infection therefore have no consolidated account of how many patients have been treated, with which regimens, with what outcomes and toxicity, and how far the reports overlap. Guidance now exists for synthesising heterogeneous single-arm and case-level evidence without meta-analysis [42,43]. The objective of this review was to identify, appraise and synthesise all human studies of fosmanogepix used to treat invasive or otherwise serious fungal infections that could be identified from the searched sources. The research question was: in patients of any age with a proven, probable or clinically diagnosed fungal infection treated with fosmanogepix alone or in combination, with or without a comparator, what treatment responses, survival, and adverse events have been reported across all study designs?

## 2. Materials and Methods

### 2.1. Review Design, Protocol and Reporting Guidelines

This systematic review with structured narrative synthesis followed the PRISMA 2020 statement [43] and the Synthesis Without Meta-analysis (SWiM) guideline [42]. The question was framed with population, intervention, comparator, and outcome elements: patients of any age with an invasive or otherwise serious fungal infection (population) who received at least one dose of fosmanogepix as treatment (intervention), with or without any comparator (comparator), and any clinical, mycological, radiological, survival, or safety outcome (outcomes). All designs were eligible, from prospective single-arm trials to case reports, congress abstracts and preprints, because a scoping search showed that the largest treated populations have been reported only as abstracts and that restriction to full articles would exclude most treated patients.

A protocol specifying the question, eligibility criteria, information sources, search terms, data items, appraisal tools, and synthesis plan was written before the searches were run. The review protocol was registered with the Open Science Framework (OSF; registration code: https://osf.io/b4dsh, accessed on 1 September 2026). One clarification was recorded during full-text assessment and applied uniformly: reports in which fosmanogepix was given but no clinical, mycological, radiological, or safety observations after the first dose were described were classified as ineligible because they contribute no outcome information. No ethical approval was required because only published data were used. Generative artificial intelligence was used during the conduct of the review, as disclosed in the Acknowledgements; all extracted values were verified against the source documents, and the authors take full responsibility for the content.

### 2.2. Eligibility Criteria

Studies were eligible if they met all of the following criteria. Population: human patients of any age and immune status with a fungal infection that was invasive, disseminated, deep-seated, osteoarticular, ocular with intraocular extension, or a chronic cutaneous infection in an immunocompromised host, whether proven, probable, or possible by the 2020 EORTC/MSGERC definitions or clinically diagnosed [44]. Intervention: at least one dose of fosmanogepix by any route, dose, or duration, as monotherapy or combination, and as first-line, salvage, step-down, or continuation therapy. Comparator: none required. Outcomes: at least one treatment response as defined by the authors, mortality or survival, relapse, adverse events, dose modification or discontinuation, or pharmacokinetic observations reported after the first dose. Design: trials, cohorts, expanded-access or compassionate-use series, outbreak investigations, case series, case reports, letters with patient data, congress abstracts, and preprints, in any language.

Reports were excluded if they described only in vitro, animal, or healthy-volunteer studies; evaluated fosmanogepix in patients without an established infection, such as the phase 1b study in neutropenic leukaemia [24]; mentioned the drug only as an option, susceptibility result, or prospect without administering it; administered it without reporting any subsequent outcome or safety observation; were reviews, editorials, or commentaries without original data; or duplicated a more complete report of the same patients, in which case the most complete report was the primary record and the others were companion reports used only for additional data. Records for which neither the full text nor a complete abstract could be retrieved were classified as not retrieved. No report was excluded solely for lacking a peer-reviewed full text, but the level of reporting was recorded for every report and considered in the appraisal and synthesis.

### 2.3. Literature-Search Strategy

The literature search included publications available up to 1 September 2026. PubMed/MEDLINE was searched with the strategy “fosmanogepix OR manogepix OR APX001 OR APX001A OR “PF-07842805”” (194 records) and with a broader strategy adding the historical codes E1210 and E1211 (326 records; the additional hits were preclinical or unrelated). Because abstracts, preprints and case reports in speciality journals are incompletely indexed, Google Scholar was searched with five query strings combining “fosmanogepix” with “case report”, “expanded access”, “compassionate”, “single patient”, “emergency use”, “abstract”, “poster”, “patient treated”, the names of the main target pathogens and “was treated”, plus an all-in-title search; the first 100 results of each query, or all results when fewer, were screened (539 records). ClinicalTrials.gov was searched for the intervention “fosmanogepix OR APX001” (14 records). Supplements of Open Forum Infectious Diseases, the Journal of Heart and Lung Transplantation, Bone Marrow Transplantation, the American Journal of Transplantation, Chest, Critical Care Medicine and the Journal of Burn Care & Research were searched through the publishers’ sites and Google Scholar, and the ESCMID Global 2025 abstract book was searched for fosmanogepix.

Backward citation searching was performed on the reference lists of all included reports and of recent narrative reviews, and forward citation searching through Google Scholar for the three phase 2 trials and the first case reports. Europe PMC could not be queried during the search window, and Embase, Web of Science, and Scopus were not searched because the authors had no institutional subscription to these databases at the time of the review. Several features of the search were intended to mitigate this restriction: fosmanogepix and its development codes are unique terms without synonyms, so free-text retrieval in PubMed and Google Scholar does not depend on controlled vocabulary; Google Scholar indexes the full text of the major publishers whose journals are covered by Embase and Scopus, including congress supplements; and backward citation searching of every included report and of the three most recent narrative reviews [2,40,41], together with forward citation searching, identified no eligible report that the database searches had missed. The cap of 100 results per Google Scholar query reflects its relevance ranking, in which eligible records clustered in the first pages of each query, but records beyond the cap were not screened. Full texts were obtained from PubMed Central, publisher websites and repositories, and abstracts from the supplement pages of the respective journals. Records that remained inaccessible, such as a paywalled congress supplement or a registration-walled clinical picture, were classified as not retrieved rather than extracted from secondary citations, and no data were taken from press releases or review articles. Search strings, dates, and result counts were logged and are available on request.

### 2.4. Study Selection and Data Extraction

Titles and abstracts were screened against the eligibility criteria, and all potentially eligible records proceeded to full-text assessment, where eligibility was decided by consensus with reference to the original text. Multiple reports of the same population were identified by comparing authors, institutions, enrolment periods, registration numbers, and patient descriptions. The two 2020 IDWeek abstracts of the candidaemia trial were linked to the full publication [27,45], the commentary on the first *Aspergillus calidoustus* case to the case report [46,47], the 2023 surgical report of a lung-transplant recipient to the congress abstract that named the drug [48,49], and the three reports of the Matamoros *Fusarium* meningitis outbreak were treated as one cohort with the 2026 investigation as the primary record [32,50,51]. Overlap between the expanded-access abstracts and other reports could not be resolved and is flagged rather than eliminated. Three categories of overlap were distinguished: resolved duplicates (the companion reports listed above, which were merged into single studies); definite overlap that could not be removed without losing data (one patient in the single-centre transplant series [52] and one in the mucormycosis series [53] had also been enrolled in the phase 2 mould trial [29] and are counted in both records); and unresolvable probable or possible overlap (the eight United States outbreak patients in the fusariosis abstract [31], who probably correspond to the seven outbreak-cohort recipients [32]; the single-centre transplant series [52] and the two paediatric mucormycosis cases [54], which come from centres contributing to the programme abstracts; and individually published expanded-access cases with *Fusarium*, *Aspergillus* or Mucorales infection, which may also have been captured in the programme abstracts [31,34,53]). Because the abstracts contain no patient identifiers, these overlaps could not be verified, and their possible effect was explored in scenario analyses (Section 2.5). Throughout the review, counts outside the trials are therefore reported as patient-episodes rather than as unique patients.

Data were extracted into a piloted structured form covering bibliographic details and level of reporting; design, country, and registration; population (number receiving fosmanogepix, number analysed, age, sex, underlying conditions, immune status); infection (pathogen, site, EORTC/MSGERC category, prior antifungal therapy, in vitro susceptibility); intervention (loading dose, maintenance dose and route, oral switch, duration, concomitant antifungals, surgery, monotherapy versus combination); outcomes (primary end-point definition, response, mycological response, mortality, relapse, follow-up, adverse events, drug-related and serious events, discontinuations, pharmacokinetics); and the authors’ conclusion. Every value was copied with a supporting quotation and its location in the source. Values not reported were entered as NR; nothing was estimated, imputed or derived from figures, and percentages were recalculated only where numerator and denominator were both stated. Extraction was performed by one reviewer and verified in full by a second reviewer, who checked every extracted value and its supporting quotation against the source document; discrepancies were resolved by discussion, with the third author consulted where needed, and corrected before synthesis. Fully independent duplicate extraction was not performed, and this is acknowledged as a limitation (Section 5).

### 2.5. Quality Assessment and Evidence-Synthesis Methods

Methodological quality was appraised with the Joanna Briggs Institute (JBI) checklists: the nine-item checklist for quasi-experimental studies for the single-arm trials [55], the ten-item checklist for case series for expanded-access, outbreak and cohort series [56], and the eight-item checklist for case reports for single-patient reports and abstracts [57]. Items were rated yes, no or unclear; the two items that do not apply to single-arm designs were excluded from the denominator. The number of items met is reported in the Results (Section 3.5), and the principal concerns are summarised in the Results. No quality threshold was used to exclude studies; the appraisal and the level of reporting were used to weight the interpretation of findings.

Statistical pooling was judged inappropriate because the studies differed in pathogen, site, host, line of therapy, concomitant treatment, dose, duration, and outcome definitions; most reports concerned a single patient, and several populations overlapped. A structured narrative synthesis was therefore undertaken according to SWiM [42]. Studies were grouped by design and by pathogen, outcomes were tabulated with their original denominators and definitions, and vote counting by direction of effect was avoided. Design differences were handled explicitly in the synthesis. Outcomes were presented within four strata that were never combined into an aggregate estimate: registered single-arm trials with blinded committee adjudication; expanded-access and cohort series with physician-assessed or author-adjudicated outcomes; the outbreak cohort, which reports survival only; and case-level reports, in which the reviewers classified the last reported status. Where an outcome was reported in more than one stratum, the strata are compared descriptively, and the trial data are given interpretive precedence, because they alone combine prospective enrolment, protocol-defined follow-up, and independent adjudication; the series are treated as the main source for duration of treatment and long-term tolerability; and the case-level reports are treated as supportive evidence for activity at particular sites or against particular pathogens. Outcomes were additionally stratified by whether fosmanogepix was given as monotherapy or with a concomitant systemic antifungal. For the 21 patients described individually, the reviewers classified the last reported status as favourable, partial, indeterminate, failure or not assessable using explicit rules stated in the Results, and this classification is distinguished from the authors’ own conclusions. Because GRADE is designed for comparative effect estimates and none of the included studies had a concurrent comparator, a formal GRADE profile could not be constructed. Instead, a GRADE-informed structured certainty assessment was applied to each pathogen-indication pair (candidaemia, *C. auris* candidaemia, refractory invasive mould disease, aspergillosis, fusariosis, mucormycosis, coccidioidomycosis, and *Scedosporium* or *Lomentospora* infection) and to tolerability. Each body of evidence started at low certainty, as for observational designs, and was downgraded for risk of bias, inconsistency, indirectness, imprecision, or suspected publication bias; no upgrading criteria were applied. The resulting ratings of low or very low certainty and the reasons for downgrading are reported in Section 3.6 and are intended to convey the confidence with which each finding can be applied clinically, not to replace the narrative appraisal.

## 3. Results

### 3.1. Study Selection

The searches identified 755 records: 194 from PubMed, 539 screened results from the five Google Scholar queries, 14 from ClinicalTrials.gov, and 8 from congress supplements, company listings and citation searching (Figure 1). Google Scholar results were screened on screen and duplicates across sources were recognised at the title stage, so the number of duplicate records was not quantified; every record was screened once. Fifty-seven reports were sought in full, and three could not be retrieved: an 85-patient fusariosis cohort in a paywalled journal whose abstract does not confirm that any patient received fosmanogepix, the answer section of a Clinical Infectious Diseases photo quiz describing compassionate-use fosmanogepix for fungal septic arthritis in a lung-transplant recipient, and a registration-walled clinical picture of disseminated fusariosis after stem-cell transplantation. Two ESCMID Global 2025 abstracts and one EBMT 2025 abstract were obtained from the congress abstract books.

Fifty-four reports were assessed for eligibility, and 20 were excluded: ten in which fosmanogepix was discussed, requested or tested in vitro but never given [58,59,60,61,62,63,64,65,66,67]; five in which it was given but no subsequent outcome was reported [68,69,70,71,72]; one phase 1b study in neutropenic leukaemia patients without established infection [24]; three duplicate or secondary reports without new patient data [45,46]; and one company press release. Three companion reports of the Matamoros outbreak cohort were retained as one study [32,50,51], the 2023 surgical report of a lung-transplant recipient, which does not name the drug but documents one-year survival, was retained as a companion of the corresponding abstract [48,49], and the ESCMID Global 2025 abstract of the fusariosis expanded-access programme (81 patients, October 2018 to October 2024) was retained as a companion of the later IDWeek 2025 report of the same programme (92 patients) [31,73]. In total, 30 studies in 34 reports were included: three phase 2 trials, seven expanded-access, outbreak or cohort series, and 20 case reports or single-patient abstracts. The reports comprised 22 full articles (one, Cobo et al., accessible only as an abstract), eleven congress abstracts, and one preprint, published between 2022 and 2026.

### 3.2. Characteristics of the Included Studies

Table 1 summarises the included studies. The three trials enrolled 51 participants, and each had a distinct population: older non-neutropenic adults with candidaemia, nearly half in intensive care and two thirds with renal impairment [27]; younger critically ill trauma and burn patients with *C. auris* candidaemia in two South African units [28]; and a haematological population with invasive mould disease, 20 of 21 with acute leukaemia or a related disorder [29]. Two trials were terminated early, one because of pandemic-related recruitment failure and one to prioritise a randomised phase 3 study, which explains the small denominators [28,29]. The expanded-access abstracts contribute the largest numbers but the least detail. They report 24 patients with invasive aspergillosis and 92 with *Fusarium* infection from seven countries, with a data cut-off of 1 March 2025, and rely on prospective but unmonitored physician-completed forms [31,34]. The aspergillosis series was 71% haematological, with five solid-organ transplant recipients, and 8 of 17 haematological patients had breakthrough infection on azole prophylaxis [34]. The *Fusarium* series spanned haematological malignancy, trauma, diabetes and previously healthy women infected during epidural anaesthesia, and included a ten-year-old child [31]. The mucormycosis expanded-access series described eleven patients (nine male; median age 50 years, range 15 to 69) treated between May 2021 and June 2024, seven with haematological conditions, two solid-organ transplant recipients, one with trauma and one with diabetes, and infection of the lung (5), sinus or rhinocerebral region (3), skin (2), or liver (1); one had entered expanded access after 42 days in the phase 2 trial [53]. A single-centre series identified four allogeneic transplant recipients with disseminated *Fusarium solani* infection in the first 100 days after transplantation, three with acute myeloid leukaemia, who were switched to fosmanogepix monotherapy [52]. The coccidioidomycosis series described eleven mostly immunocompetent adults, eight with meningitis [33], and the outbreak cohort comprised young, previously healthy women [32]. Two of 21 patients in a nine-year single-centre fusariosis cohort received fosmanogepix, both with acute myeloid leukaemia [74].

The 20 case-level reports describe 21 patients aged 10 to 75 years, including four children or adolescents [36,54,76]; 14 were male, five female and two of unstated sex. Nine had haematological malignancy with or without transplantation [35,37,47,75,77,78,81,82,83], five were solid-organ transplant recipients [49,79,84,87,88], one had primary immunodeficiency with kidney failure [36], two had diabetic ketoacidosis [54], one had severe burns [85], one had end-stage renal disease with multiple comorbidities [86], and two had no immunocompromise beyond trauma or corticosteroids [76,80]. Seventeen case-level reports came from the United States, where emergency investigational and expanded-access pathways are most accessible; single reports came from Greece and Spain, and one preprint did not state its country. Approximately 223 patient-episodes were described: 51 in trials, 151 in series and 21 in case-level reports (Figure 2). *Fusarium* spp. accounted for at least 114 patient-episodes, *Candida* spp. for 32 (11 with *C. auris*), *Aspergillus* spp. for 25, Mucorales for 13, *Coccidioides* spp. for 11, *Scedosporium* or *Lomentospora* spp. for 6 and *Cladophialophora bantiana* for 1, with 21 trial participants with mixed or unidentified moulds. This total contains overlap: the fusariosis abstract includes eight United States outbreak patients who probably overlap with the seven outbreak-cohort recipients; the single-centre transplant series and the two paediatric mucormycosis cases come from centres that contributed to the expanded-access abstracts, and one patient in the transplant series and one in the mucormycosis series had also been enrolled in the phase 2 trial; and individually published expanded-access cases were probably also captured in the programme abstracts.

Under the duplication scenarios (Appendix A, Table A1), the estimated number of unique patients was 214 after removal of the two patients enrolled in both a trial and a series and of the seven outbreak recipients probably included in the fusariosis abstract, 197 under the maximal-overlap assumption, and 206, 189 and 171 under uniform duplication rates of 10%, 20% and 30% among the 172 non-trial patient-episodes. The corresponding ranges were 80 to 106 unique patients for *Fusarium* spp. (source-reported, 114), 18 to 25 for *Aspergillus* spp. (source-reported, 25) and 9 to 12 for Mucorales (source-reported, 13); counts for *Candida* spp., *Coccidioides* spp. and *Scedosporium* or *Lomentospora* spp. were affected only by the uniform scenarios, because no overlap was identified for these pathogens. The total of approximately 223 should therefore be read as an upper bound on the number of individuals treated, with a plausible range of about 170 to 215.

### 3.3. Infections Treated and Fosmanogepix Regimens

Table 2 summarises the infections and regimens. Fosmanogepix was first-line therapy only in the candidaemia trial, which allowed at most two days of prior antifungal treatment, and in one case report in which it was started together with liposomal amphotericin B at presentation [27,82]. The *C. auris* trial required prior echinocandin exposure and limited treatment options [28]. In every other study, the drug was salvage or second-line therapy after failure of, intolerance to, or in vitro resistance to conventional agents, usually liposomal amphotericin B and one or more triazoles, and in five patients after olorofim [33,49]. In the mucormycosis series, ten of eleven patients had received liposomal amphotericin B, isavuconazole or posaconazole and continued or de-escalated these agents alongside fosmanogepix [53], whereas the four transplant recipients with fusariosis were switched from amphotericin B-based regimens to fosmanogepix monotherapy after a median of 8 days [52]. Reported manogepix values were uniformly low: 0.002 to 0.03 µg/mL for *Candida* spp. [27,28], 0.008 to 0.03 µg/mL for the tested moulds in the trial [29], and 0.008 to 0.015 µg/mL in the individually reported *Fusarium*, *A. calidoustus* and *L. prolificans* cases, whereas voriconazole and posaconazole values were typically 8 to above 16 µg/mL and amphotericin B 1 to 2 µg/mL [35,36,37,47,74,75,78,80]. The exceptions were a *Mucor circinelloides* isolate in the trial (4 µg/mL) and a *Rhizopus* isolate in a child (2 µg/mL) [29,54].

Dosing followed the phase 1 regimen of 1000 mg intravenously twice on day 1, 600 mg intravenously once daily and oral maintenance with 700 mg (candidaemia trial) or 800 mg once daily, with the oral switch allowed from day 4 [27,28,29]. Among the 21 individually described patients, intravenous loading was documented in five and omitted in five, with the remainder not reported (Figure 3) [35,36,37,47,54,75,76,78,80]; nine of eleven coccidioidomycosis patients and eight of eleven mucormycosis patients also started directly on oral 800 mg daily [33,53]. Splitting the daily dose into two administrations for tolerability was used in six of 21 trial participants with mould disease and in three case reports [29,36,37,76]. Treatment duration diverged sharply between trials and real-world use: 14 days at most in the candidaemia trial and 42 days in the other two trials, compared with medians of 69 to 112 days and up to 625 days in expanded access [31,34,73], a median of 89 days in mucormycosis [53], a median of 208 days and up to 1086 days in coccidioidomycosis [33], and six to ten months or longer in individual patients, in one case indefinitely [36,47,54,75,76,82]. Monotherapy was the rule in the trials and in localised infections, whereas most disseminated mould infections and both paediatric mucormycosis cases were treated with combinations, most often liposomal amphotericin B or isavuconazole, and with surgery [47,54,76,78]. Concomitant therapy was not reported for the two largest expanded-access series, so the contribution of fosmanogepix itself to the responses in those 116 patient-episodes cannot be judged.

### 3.4. Clinical Outcomes and Safety

Table 3 presents the outcomes. In candidaemia, adjudicated success at the end of study treatment was 16 of 20 (80%; 95% CI 56.3–94.3) in the mixed-species trial and 8 of 9 (88.9%; 95% CI 51.8–99.7) in the *C. auris* trial, with Day-30 survival of 85% and 89%. Sustained success four weeks after treatment fell to 55% and 67% because of relapses and deaths from underlying disease, and blood cultures cleared after a mean of 2.4 and 8.7 days in the small subsets with positive pre-treatment cultures [27,28]. In invasive mould disease, Day-42 mortality was 5 of 20 (25%; 80% CI 12.7–41.5), which met the pre-specified comparison with a historical rate of 45%, but adjudicated global response was only 8 of 20 (40%) with four complete responses, and Day-84 mortality reached 40% [29]. Physician-assessed favourable responses in expanded access were 13/17 (76%) and 4/7 (57%) in haematological and non-haematological aspergillosis, with six-week mortality of 24% and 43% [34], and 38/50 (76%) and 26/32 (81%) in haematological and non-haematological fusariosis, with 32% of haematological patients dying or entering hospice within six weeks and no early deaths among the others [31].

The earlier ESCMID data cut of the fusariosis programme gave similar figures: 34/44 (77.3%) and 32/37 (86.5%) favourable responses, 6-week mortality of 14/44 (31.8%) in haematological patients, and 9/10 favourable responses in outbreak meningitis [73]. In mucormycosis, 7/11 (64%) had a favourable clinical or radiological response, two patients died after 14 and 156 days of therapy, and two stopped for nausea or vomiting after 9 and 15 days [53]. All four transplant recipients with disseminated fusariosis had mycological and radiological responses on monotherapy, with no deaths at 6 or 12 weeks; one stopped at day 38 for nausea and vomiting [52]. Adjudicated success in refractory coccidioidomycosis was 8/11 (73%), including 6/8 with meningitis [33]. In the outbreak cohort, 6 of 7 recipients survived compared with 3 of 11 antifungal-treated non-recipients, a difference that the investigators attributed in part to later treatment and accumulated experience [32,50].

For the 21 individually described patients, the reviewers classified the last reported status as favourable in 16, partial in one, indeterminate in two, failure in one and not assessable in one. Favourable denoted resolution or sustained improvement with survival at last report, or death from an unrelated cause after documented resolution; partial denoted improvement at some sites with persistence elsewhere; indeterminate denoted findings that could not be read as response or failure; failure denoted progression on therapy; and not assessable denoted two days of therapy or less. Seventeen patients were alive at last report. Four died: one of progressive *L. prolificans* meningitis on fosmanogepix [35], one of bacterial sepsis with persistent pulmonary fusariosis after four days of therapy [81], one of relapsed leukaemia after resolution of disseminated *L. prolificans* infection [78], and one after electing hospice two days into therapy [79]. Follow-up after stopping fosmanogepix reached or exceeded one year in five patients without relapse [54,75,80,87], and a possible relapse two months after a three-month course of monotherapy was retreated successfully [37]. Of the two cohort patients, one had a partial response and survived 963 days, and the other did not respond and died on day 41 [74]. Sustained cures of central nervous system and ocular fusariosis, of disseminated aspergillosis with endocarditis and of cerebral phaeohyphomycosis were documented, some without surgery [47,75,80,87].

Table 4 stratifies the reported outcomes by study design and by concomitant systemic antifungal therapy; no estimate was pooled across strata. Monotherapy was mandated in the three trials, in which all adjudicated outcomes are therefore attributable to fosmanogepix alone, and was used in the four transplant recipients with disseminated fusariosis, all of whom responded [52]. In contrast, the mucormycosis series (ten of eleven patients on continued amphotericin B or an azole), the coccidioidomycosis series (at least eight of eleven on concomitant therapy), the outbreak cohort and the two-patient fusariosis cohort all combined fosmanogepix with other systemic agents, and concomitant therapy was not reported for the two largest series, so the favourable responses in those 116 patient-episodes cannot be attributed to fosmanogepix alone [31,32,33,34,53,74]. Among the 21 individually described patients, nine received a concomitant systemic antifungal, most often liposomal amphotericin B or isavuconazole, seven received fosmanogepix as the only systemic antifungal (two with additional intraocular or intracavitary agents only), and five reports did not state concomitant therapy.

Favourable outcomes were recorded in seven of nine patients treated with combinations, in five of seven treated with monotherapy and in four of five with unreported co-therapy; the single clear failure, the fatal *L. prolificans* meningitis, occurred on monotherapy, whereas both paediatric mucormycosis responses and the cures of disseminated *A. calidoustus* and *L. prolificans* infection occurred with combination therapy and surgery [35,47,54,78]. Responses to fosmanogepix as the sole systemic agent were therefore documented mainly in localised or cutaneous *Fusarium* infection, in keratitis with anterior-chamber involvement, in cerebral phaeohyphomycosis and in the transplant fusariosis series, whereas most disseminated mould infections that responded had been treated with combinations and surgery, and the independent contribution of the drug in those patients cannot be determined [36,37,52,80,84,87].

Safety data were most complete in the trials. Drug-related adverse events occurred in 1 of 21 candidaemia participants (moderate thrombocytopenia), in none of nine *C. auris* participants and in 15 of 21 (71%) mould-disease participants, in whom nausea (52%), vomiting (33%) and diarrhoea (24%) predominated. No participant in the candidaemia trials stopped for a drug-related event, whereas 7 of 21 (33%) with mould disease stopped for any adverse event and 3 (14%) for drug-related events, all gastrointestinal and none severe [27,28,29]. One unwitnessed sudden death on day 14 in the mould trial was judged possibly related by the investigator and unrelated by the monitoring board; autopsy showed invasive aspergillosis with haemorrhage [29]. No haematological, biochemical, electrocardiographic or renal signal was identified, and manogepix exposure was not increased in renal impairment [27]. In expanded access, gastrointestinal events caused three discontinuations among 92 fusariosis patients (three among 81 in the earlier data cut), two of eleven mucormycosis patients and one of four transplant recipients with fusariosis stopped for nausea or vomiting, and one aspergillosis patient stopped for a serious central nervous system event with altered mentation, extrapyramidal symptoms and seizures [31,34,52,53,73], and grade 1 to 3 nausea or vomiting affected 6 of 11 coccidioidomycosis patients treated for up to three years [33]. In the case-level literature, gastrointestinal intolerance was reported in seven patients and managed with dose splitting, food, or antiemetics [35,36,37,54,76,79,81]; suspected neurotoxicity was described once and remained unconfirmed [35]; and no hepatic, renal or cardiac toxicity attributable to the drug was reported during courses of up to 18 months, including in a patient on peritoneal dialysis and in two children [36,54,82].

Hepatic adverse events, discontinuations and drug interactions were examined specifically because they are the usual limiting toxicities of systemic antifungals. No hepatic adverse event attributed to fosmanogepix was reported in any included study. In the three trials, liver function was monitored as part of the protocol-defined safety laboratory assessments, and no drug-related elevation of transaminases or bilirubin was identified, including during 42 days of treatment in the mould-disease trial [27,28,29]; in the expanded-access series and case reports, hepatic laboratory values were reported only sporadically, so the absence of a hepatic signal outside the trials reflects incomplete reporting rather than demonstrated safety.

Discontinuation for adverse events was documented in 3 of 51 trial participants for drug-related events (all gastrointestinal, all in the mould trial) and in 7 of 51 for any adverse event, and in 7 of 131 patient-episodes in the four series that reported this outcome (3 of 92 with fusariosis, 1 of 24 with aspergillosis, 2 of 11 with mucormycosis and 1 of 4 in the transplant series), with gastrointestinal intolerance the reason in all but the single central nervous system event; discontinuation was not reported for the coccidioidomycosis series or the outbreak cohort [29,31,33,34,52,53]. In the case-level reports, one patient interrupted fosmanogepix for six days for suspected neurotoxicity and one stopped on entering hospice, and no other discontinuation for toxicity was described [35,79]. No clinically relevant drug interaction was reported in any included study, although none assessed interactions formally: fosmanogepix was given with liposomal amphotericin B, isavuconazole, posaconazole, or voriconazole, with immunosuppressive regimens in transplant recipients and during peritoneal dialysis, without any reported change in toxicity or, where measured, in manogepix exposure [33,36,47,53,54,76,79,88], and phase 1 studies had characterised a modest interaction profile [23]. Manogepix concentrations were measured in only a few case reports, so interaction-related changes in exposure would not have been detected.

### 3.5. Methodological Quality of the Included Studies

Methodological quality is summarised in the last column of Table 3 and in Figure 4, which shows the proportion of applicable checklist items met, unclear, or not met for each study. The three trials met every applicable JBI criterion except for a control group, used blinded independent adjudication and reported follow-up completely, but each was industry-sponsored and small, and the mould trial relied on a historical benchmark and could not identify the pathogen in 12 participants [27,28,29]. The expanded-access abstracts met five or six of ten case-series items and were unclear for the rest, because inclusion beyond ‘completed fosmanogepix treatment’, consecutive enrolment, completeness, and the definition of documented infection were not stated, and outcomes were physician-reported without monitoring [31,34,53]. The single-centre transplant series met nine items, being drawn from a microbiology database with defined dates, but reported no dosing and only 12 weeks of follow-up [52]. The coccidioidomycosis series met nine of ten items, its weaknesses being retrospective author adjudication and extensive concomitant therapy [33]. The outbreak cohort was rigorous at cohort level but reported neither dosing nor adverse events for the fosmanogepix subgroup, and it is exposed to immortal-time bias because the drug became available months after the first exposures [32,50]. Eight of 13 full-article case reports met all eight items [35,36,37,47,54,75,76,80], whereas the congress abstracts met between two and six, most often lacking dose, duration, susceptibility values or adverse events [49,84,85,86,87,88].

Publication bias is a pervasive concern: 16 of 21 individually reported patients had favourable outcomes, only one report was written to document failure, and its authors cautioned that favourable results are over-represented [35,81]. Formal tests for publication bias could not be applied because they require effect estimates with standard errors from comparative studies; the reporting-bias scenario (Appendix A, Table A3) indicates that the published case-level literature would be consistent with 4 to 19 unpublished failures if the true favourable proportion resembled the physician-assessed rate in mucormycosis or the adjudicated rate in the mould trial. Adverse-event reporting was similarly uneven: drug-attributed adverse events were reported in 19 of 30 studies, severity grading in the three trials and the coccidioidomycosis series, and formal causality assessment only in the trials and most full case reports, whereas the expanded-access abstracts and most single-patient abstracts gave no adverse-event data beyond discontinuations (Table 3). Statements about response rates in this review therefore rest on the trials and on the adjudicated coccidioidomycosis series; statements about long-term tolerability rest on the expanded-access series and full case reports, and the abstract-only reports are treated as supportive.

### 3.6. Certainty of Evidence by Indication

Table 5 summarises the GRADE-informed certainty assessment for each pathogen-indication pair. Certainty was rated low for adjudicated treatment success in candidaemia and for Day-42 mortality and global response in refractory invasive mould disease, because these outcomes come from registered prospective trials with blinded adjudication and complete follow-up, but they were downgraded for very serious imprecision (20 or fewer evaluable participants) and, for the mould trial, for indirectness arising from the unidentified pathogen in 12 participants and the historical benchmark. Certainty was very low for *C. auris* candidaemia (nine trial participants and two abstracts), for aspergillosis, fusariosis, and mucormycosis (unmonitored physician-assessed outcomes in abstracts, unresolved overlap, unreported concomitant therapy, and suspected reporting bias), for coccidioidomycosis (a single retrospective series with author adjudication and extensive co-treatment) and for *Scedosporium* or *Lomentospora* infection (six case-level reports including one failure). For fusariosis, the direction of effect was consistent across the programme abstracts, the transplant series, the outbreak cohort and nine case reports, which was noted but did not raise the rating, because consistency among sources that share the same biases is not evidence of validity. Tolerability during prolonged therapy was rated low, on the basis of the trials supplemented by the series with months to years of exposure. These ratings mean that further evidence, in particular the phase 3 trials, is likely to change the estimates for every indication.

## 4. Discussion

This review assembled all identifiable human evidence from the searched sources on fosmanogepix as treatment for fungal infection: 30 studies in 34 reports describing approximately 223 reported patient-episodes (an estimated 171 to 214 unique patients), of whom only 51 were enrolled in prospective trials. The findings are consistent across designs. In candidaemia, including *C. auris* candidaemia, independent adjudication gave end-of-study-treatment success of 80% and 89% and Day-30 survival of 85% and 89%, with no drug-related discontinuations [27,28]. In refractory invasive mould disease, Day-42 mortality of 25% was lower than the historical benchmark, but only 40% of participants achieved an adjudicated response, and one third stopped therapy for adverse events [29]. In expanded access, physicians judged responses favourable in about three quarters of patients with aspergillosis or fusariosis, in 7 of 11 with mucormycosis and in 8 of 11 with refractory coccidioidomycosis, and treatment continued for months to years [31,33,34,53]. At the individual level, 16 of 21 patients whose infections had failed licensed agents, mostly *Fusarium* and *Lomentospora* infections in immunocompromised hosts, had favourable outcomes, and prolonged oral therapy was tolerated with mainly gastrointestinal effects. These observations should be read as signals of antifungal activity rather than as evidence of clinical effectiveness. An observed response rate in a single-arm study describes what happened to selected patients who received the drug, often alongside other agents and surgery, whereas clinical effectiveness requires a comparison with what would have happened under standard therapy. No included study provided such a comparison, so the response rates reported here, however high, cannot establish that fosmanogepix is superior, equivalent or inferior to liposomal amphotericin B, the triazoles or the echinocandins in any indication, and the GRADE-informed certainty assessment yielded ratings of low or very low for every indication (Table 5).

Viewed together, the study-level outcomes show a consistent pattern rather than a single effect size. Adjudicated success rates in candidaemia were within the range expected for echinocandin first-line therapy in similar populations, and the *C. auris* result is notable because most isolates carried fluconazole resistance and all patients had failed or could not receive standard options [27,28,89]. In refractory invasive mould disease, mortality at Day 42 was lower than the historical benchmark, but response was achieved in fewer than half, which suggests that survival benefit and disease control can diverge when a fungistatic drug is given to profoundly immunosuppressed hosts [29]. In the expanded-access series, favourable responses of roughly two-thirds to four-fifths across aspergillosis, fusariosis, mucormycosis and coccidioidomycosis, with early mortality concentrated in haematological patients, are compatible with meaningful activity in infections that carry historical mortality above 50%, but they were assessed by treating physicians in selected survivors [31,33,34,53]. The outbreak cohort, in which survival was far higher among recipients than among patients treated without the drug, provides the only comparison, and it is confounded by timing [32]. Among the 21 individually described patients, 16 had favourable outcomes, and only one clear failure was published, a distribution that almost certainly overstates the true proportion of favourable outcomes. Case reports are written to document success; expanded-access abstracts report only patients who completed treatment and rely on the judgement of the physicians who chose to use the drug; and the failures that occur in patients who die early or whose treatment is abandoned are the least likely to be written up. The reporting-bias scenario in Appendix A, Table A3 shows that the published case literature would be consistent with as many as 19 unpublished failures if the true favourable rate resembled the adjudicated trial rate, so the favourable proportions from case reports and expanded access should be regarded as upper bounds rather than as estimates. None of these findings is a statistical estimate, and no pooled effect was derived.

Comparison across studies also exposes inconsistencies that temper enthusiasm. The 40% adjudicated response in the mould trial contrasts with physician-assessed responses of 57% to 81% in expanded access for the same pathogens. The trial population was almost entirely haematological with probable disease of uncertain aetiology, whereas expanded-access patients were selected by clinicians, assessed without independent review and reported only after completing treatment, so case mix, outcome definition and survivorship probably all differ [29,31,34]. The expanded-access abstracts are further limited by the absence of independent monitoring, of validated case definitions and of standardised outcome adjudication, and by incomplete reporting of concomitant antifungals and surgery, so that unmeasured co-interventions, selection of patients by treating clinicians, survivorship (only patients who completed treatment were reported) and differences in case mix all plausibly contribute to the gap between the 40% adjudicated and the 57% to 81% physician-assessed responses. The available data do not allow the size of any one of these contributions to be estimated, and the gap should not be read as evidence that either figure is wrong; it shows only that the two sets of figures measure different things in different patients. The outbreak cohort illustrates confounding by time: recipients were those who survived until the drug became available and who were treated when clinicians already had experience of the syndrome, as the investigators noted [32,50]. Susceptibility did not predict outcome in every case. Fatal progression of *L. prolificans* meningitis occurred despite a minimum effective concentration of 0.008 µg/mL or less; one of two cohort patients with identical values did not respond, and a *Rhizopus* infection improved despite a value of 2 µg/mL, albeit with isavuconazole and extensive surgery [35,54,74]. Conversely, sustained cures of central nervous system, ocular and endovascular disease are consistent with the wide tissue distribution shown preclinically [21,47,75,80,87]. Dosing practice also varied: omission of intravenous loading, which delays steady state by one to two weeks, was common in expanded access and was raised as a possible contributor to one fatal failure [14,33,35].

The clinical implications are provisional. For candidaemia, the trial data justify the ongoing phase 3 comparison with caspofungin followed by fluconazole, and the *C. auris* data support consideration of its use where echinocandin resistance or intolerance leaves no licensed option, with the caveat that the trial excluded deep-seated infection and limited therapy to 14 days [25,27,28,89]. For *Fusarium*, *Lomentospora* and other intrinsically resistant moulds, in which guidelines acknowledge poor outcomes with voriconazole and lipid amphotericin B [3,4], the expanded-access experience of more than 90 reported patient-episodes with physician-assessed favourable responses in about three quarters, and the consistent direction of the smaller series and case reports, together with several cures in refractory disseminated disease, make fosmanogepix one of the more extensively documented investigational options, and the oral formulation permits long outpatient courses that amphotericin B cannot provide. Olorofim, the other investigational agent available through expanded access for rare moulds, has limited activity against *Fusarium* spp. but is highly active against *Lomentospora* and *Scedosporium* spp., so the choice between the two will depend on pathogen, site, and access rather than on comparative data, which do not exist [2,40]. For mucormycosis, the evidence comprises eleven expanded-access patients with a 64% favourable response, two paediatric cases, and one mixed infection, nearly all treated with continued amphotericin B or azoles and often with surgery, and for coccidioidomycosis, eleven patients treated after multiple prior agents; no recommendation beyond expanded-access use can be derived for either indication [33,53,54,88,90]. Clinicians using the drug should anticipate nausea and vomiting, consider intravenous loading when rapid exposure is needed, and use dose splitting or antiemetics rather than dose reduction for intolerance, as was done in the trials and several case reports [29,36,37].

Two questions follow for clinical positioning. The first is whether fosmanogepix has a role earlier than salvage. The only first-line data are the candidaemia trial and one case report [27,82]; every other use was after failure of, intolerance to or resistance to licensed agents. For candidaemia, the ongoing phase 3 trial will answer this directly [25]. For moulds, earlier use is conceptually attractive in infections with intrinsic resistance to licensed agents, in which conventional therapy is expected to fail, but the 40% adjudicated response and 33% discontinuation rate in the mould trial argue against displacing amphotericin B or a triazole as first-line therapy for susceptible pathogens on the present evidence, and the drug should currently be regarded as a salvage or expanded-access option outside trials. The second is which populations warrant further evaluation. On the basis of this review, the populations in whom further evaluation may be particularly relevant are patients with *Fusarium*, *Lomentospora*, or *Scedosporium* infection that is refractory to or unsuitable for licensed agents; patients who need months of oral therapy that amphotericin B cannot provide, such as those with central nervous system, osteoarticular, or ocular disease; and patients with *C. auris* candidaemia when echinocandins cannot be used. Randomised or comparative trials are needed most urgently in fusariosis and other intrinsically resistant mould infections, where the largest uncontrolled experience exists, historical mortality exceeds 50%, and no licensed agent has proven efficacy, so that a comparison with best available therapy, most plausibly voriconazole with or without lipid amphotericin B, would be both ethical and informative; refractory coccidioidal meningitis and *C. auris* infection with echinocandin resistance are the next priorities. Within such trials, stratification by haematological status and by concomitant surgery would address the two variables that most affected outcomes in the present data.

Paediatric evidence deserves separate mention because it is minimal. The included reports describe six children or adolescents: a 14-year-old with primary immunodeficiency and chronic cutaneous *Fusarium* infection treated for about six months [36], a 17-year-old with disseminated fusariosis after trauma [76], a 15-year-old and a 10-year-old with rhino-orbital-cerebral mucormycosis and diabetic ketoacidosis [54], a ten-year-old in the fusariosis expanded-access series [31] and a 15-year-old in the mucormycosis series [53]. All received adult doses, because no weight-based regimen has been defined and no paediatric pharmacokinetic data have been published; outcomes were favourable in the four individually described children, gastrointestinal intolerance was managed by dose splitting in two of them, and no growth, developmental or organ toxicity was reported over follow-up of up to two years [36,54,76]. No trial has enrolled children, no patient younger than ten years has been reported, and the evidence is therefore insufficient to support paediatric dosing recommendations; a paediatric pharmacokinetic study should accompany the phase 3 programme.

Heterogeneity was clinical (pathogen, site, host, line of therapy, concomitant treatment), methodological (adjudicated versus physician-reported versus narrative outcomes; follow-up from 30 days to more than two years), and in the level of reporting, and it precluded quantitative pooling. These differences shaped the synthesis in three ways. Outcomes were never combined across strata, because an adjudicated response in a trial, a physician-assessed response in an abstract and a reviewer classification of a case report measure different things; the trials were given interpretive precedence for response rates, the series for duration and tolerability and the case reports for site-specific activity; and each statement in this Discussion is anchored to the stratum from which it derives. Readers should therefore treat the design of the source, rather than the size of the proportion, as the primary guide to its weight. The conflicting findings, high physician-reported responses against modest adjudicated responses, and susceptibility without response, more likely reflect these differences and selective reporting than true biological inconsistency, but they cannot be resolved with the available data. Future research should prioritise completion of the two randomised phase 3 trials, which will provide the first comparative estimates [25,26]; full publication of the expanded-access programme with patient-level data on dosing, concomitant therapy, adjudicated outcomes and adverse events, and with linkage to previously published cases so that overlap can be quantified; collection of manogepix pharmacokinetic data in central nervous system, ocular and osteoarticular disease and in children, for whom weight-based dosing is undefined [36,54]; investigation of the discordance between in vitro susceptibility and response [35,74]; and deliberate publication of treatment failures. Stringent eligibility criteria exclude most patients with invasive candidiasis from trials [91], which strengthens the case for structured registries for the patients who will continue to receive fosmanogepix outside trials.

## 5. Strengths and Limitations

The strengths of this review are its broad scope, which for the first time assembles trials, expanded-access series, outbreak data, and case-level reports of fosmanogepix in a single appraised account; a registered protocol and reporting according to PRISMA 2020 and SWiM; extraction with a supporting quotation for every value; explicit rules for classifying case-level outcomes, kept separate from the source authors’ conclusions; stratification of every outcome by study design and by concomitant therapy; a GRADE-informed certainty assessment for each indication; and scenario analyses that bound the effects of patient duplication and reporting bias.

The review also has several limitations. At the evidence level, no study had a concurrent comparator; most reports concerned single patients; the largest series were available only as unmonitored abstracts without dosing or concomitant-therapy data; one full article could be read only as an abstract, and one report was a preprint. Overlap between the expanded-access abstracts, the outbreak cohort, the single-centre series and individually published cases could not be quantified, so the total of approximately 223 patient-episodes is an upper bound; the duplication scenarios suggest that between about 170 and 215 unique patients were treated, but the true figure cannot be determined without patient-level linkage, and every count and proportion outside the trials describes reported patient-episodes rather than unique individuals. Adverse events were inconsistently and incompletely reported outside the trials: severity grading was available only from the trials and the coccidioidomycosis series, causality assessment mainly from the trials and full case reports, and 11 of the 30 studies reported no drug-attributed adverse-event data at all, so uncommon toxicities, including hepatic events, cannot be excluded. Publication bias towards favourable outcomes is probable and could not be assessed with funnel-plot methods, which require comparative effect estimates; the scenario analysis indicates that the case-level literature would accommodate a substantial number of unpublished failures, and the favourable proportions from case reports and expanded access should be treated as upper bounds. The expanded-access abstracts lack independent monitoring, validated case definitions and adjudicated outcomes, and the contributions of co-interventions, selection, and survivorship to their favourable responses cannot be separated. At the review level, Embase, Web of Science, Scopus, and Europe PMC could not be searched; Google Scholar screening was capped at 100 results per query and duplicates across sources were not enumerated; three potentially relevant reports could not be retrieved, although one does not confirm that fosmanogepix was given and the other two describe single patients whose inclusion would not change the direction of any finding; extraction was performed by one reviewer with independent verification rather than fully in duplicate, which carries a higher risk of undetected extraction error than independent dual extraction; certainty was rated with a GRADE-informed approach adapted to single-arm data rather than a formal GRADE profile, so the ratings involve judgement; and the classification of case-level outcomes, although rule-based, involved judgement. Reports published after the search date, including the eventual full publication of the expanded-access programme, are not captured.

## 6. Conclusions

Across single-arm trials, expanded-access series, and case-level reports, fosmanogepix was associated with treatment success in most patients with candidaemia, including *C. auris* candidaemia, and with favourable responses in the majority of patients with fusariosis and other resistant mould infections that had failed conventional therapy, whereas adjudicated responses in refractory invasive mould disease were more modest. Gastrointestinal intolerance was the main adverse effect, and no organ toxicity attributable to the drug emerged during prolonged courses. These uncontrolled and partly overlapping data indicate signals of clinically relevant activity and acceptable tolerability, with low or very low GRADE-informed certainty for every indication, but they cannot establish comparative efficacy or superiority over standard antifungal therapy, optimal dosing or the value of intravenous loading, and efficacy in mucormycosis and central nervous system disease remains uncertain. Results of the ongoing randomised phase 3 trials and full publication of the expanded-access programme are needed before fosmanogepix can be positioned in treatment guidelines.

## Figures and Tables

**Figure 1:**
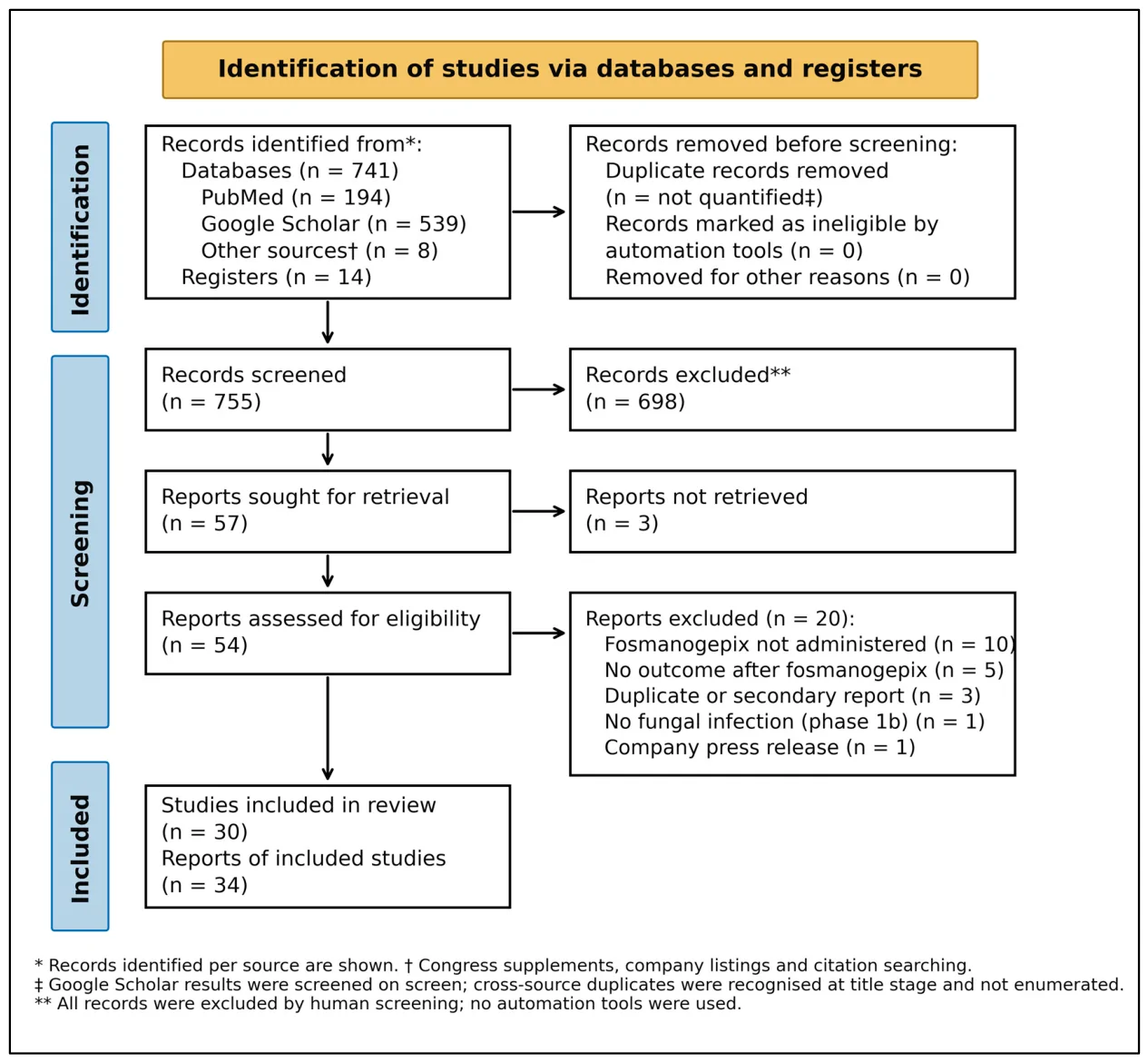
PRISMA 2020 flow diagram of study identification, screening, and inclusion [43]. Records are shown per source; duplicates across sources were recognised during title screening but not enumerated. Companion reports of the Matamoros outbreak cohort (three reports), of the fusariosis expanded-access programme (two data cuts) and of one lung-transplant case (a congress abstract and a surgical full report) account for the difference between studies and reports.

**Figure 2:**
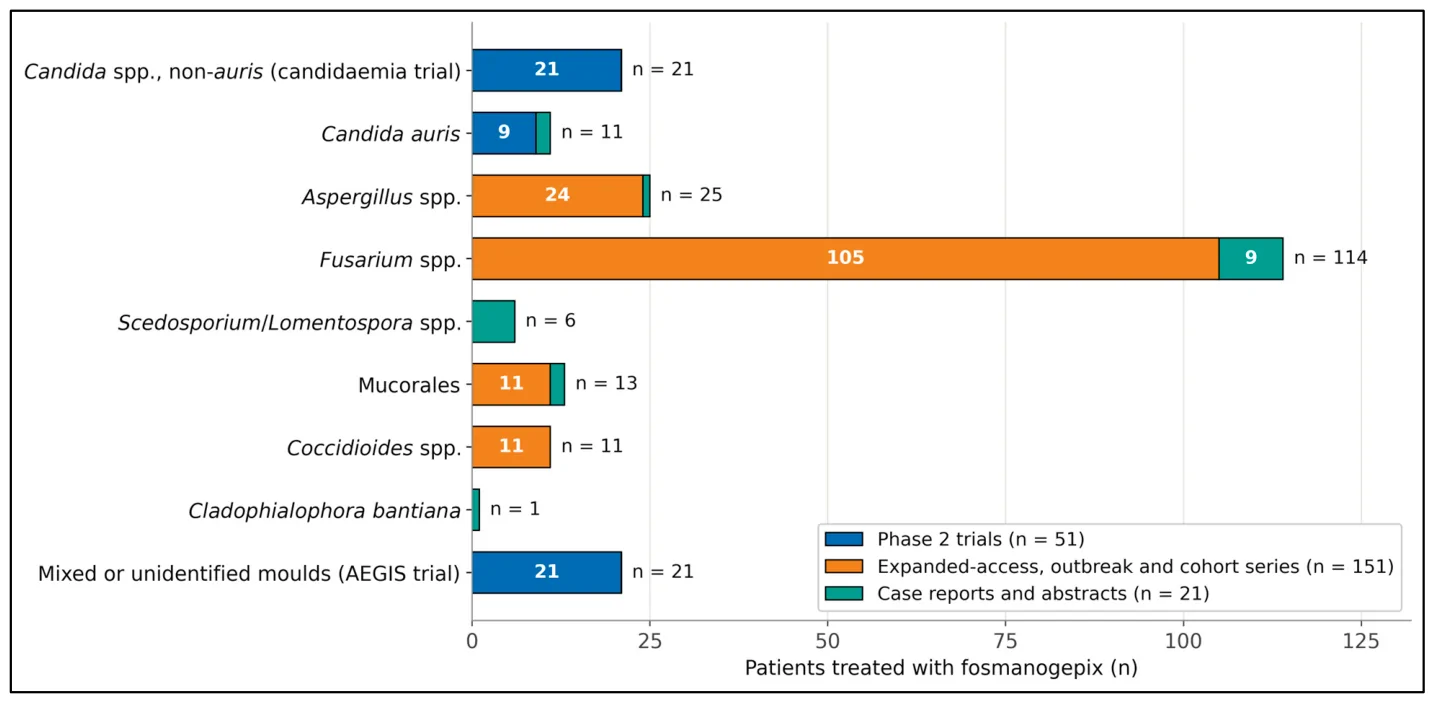
Reported patient-episodes of fosmanogepix treatment by pathogen group and study design in the included studies (approximately 223 patient-episodes). Bars are stacked by design; overlap between expanded-access series, the outbreak cohort, single-centre series and case reports cannot be excluded, so totals are upper bounds. *Candida* spp. in the candidaemia trial included *C. glabrata*, *C. albicans*, *C. parapsilosis*, and *C. dubliniensis*; the AEGIS trial enrolled *Aspergillus* spp. and rare moulds with the pathogen unidentified in 12 participants.

**Figure 3:**
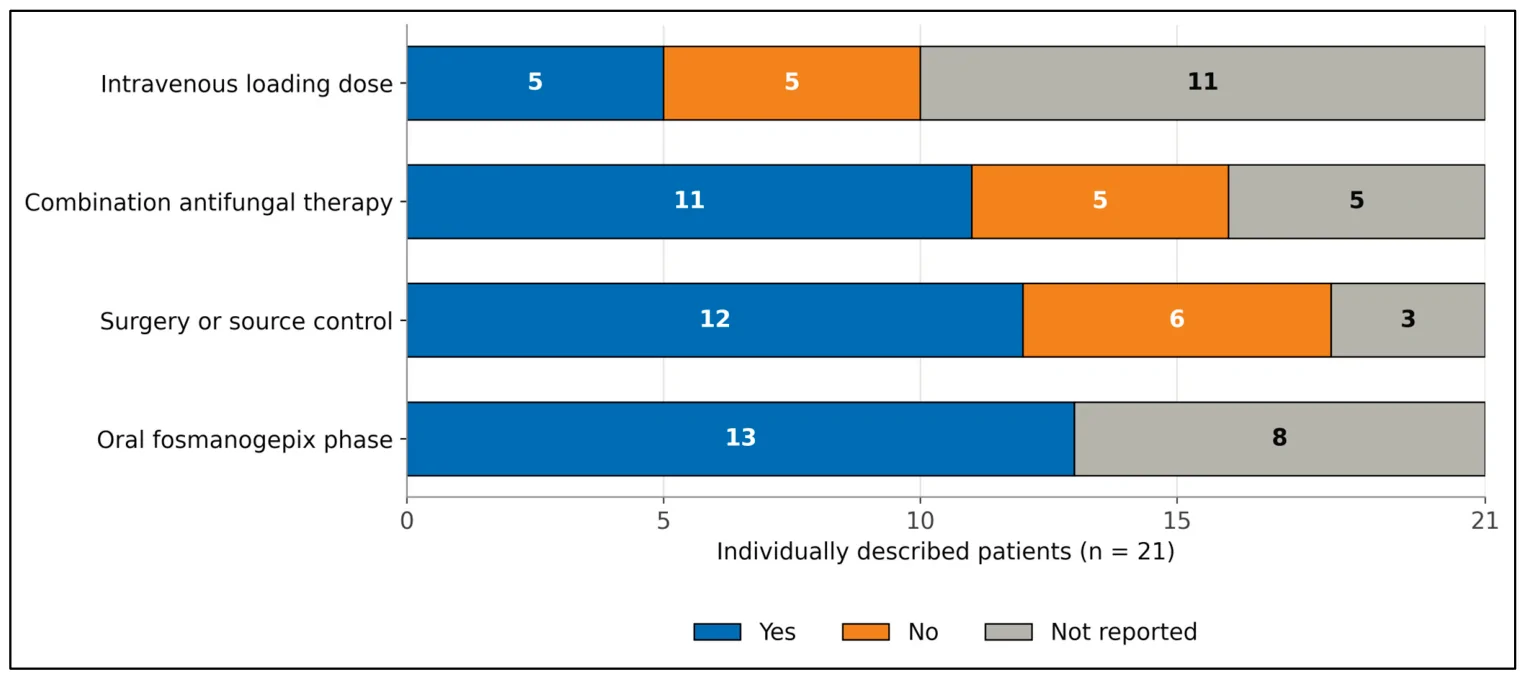
Regimen features reported for the 21 individually described patients treated with fosmanogepix in case reports and single-patient abstracts. Intravenous loading refers to 1000 mg twice on day 1; combination refers to a concomitant systemic antifungal at any time during fosmanogepix therapy; surgery includes debridement, resection or other source control; the oral phase denotes any period of oral fosmanogepix.

**Figure 4:**
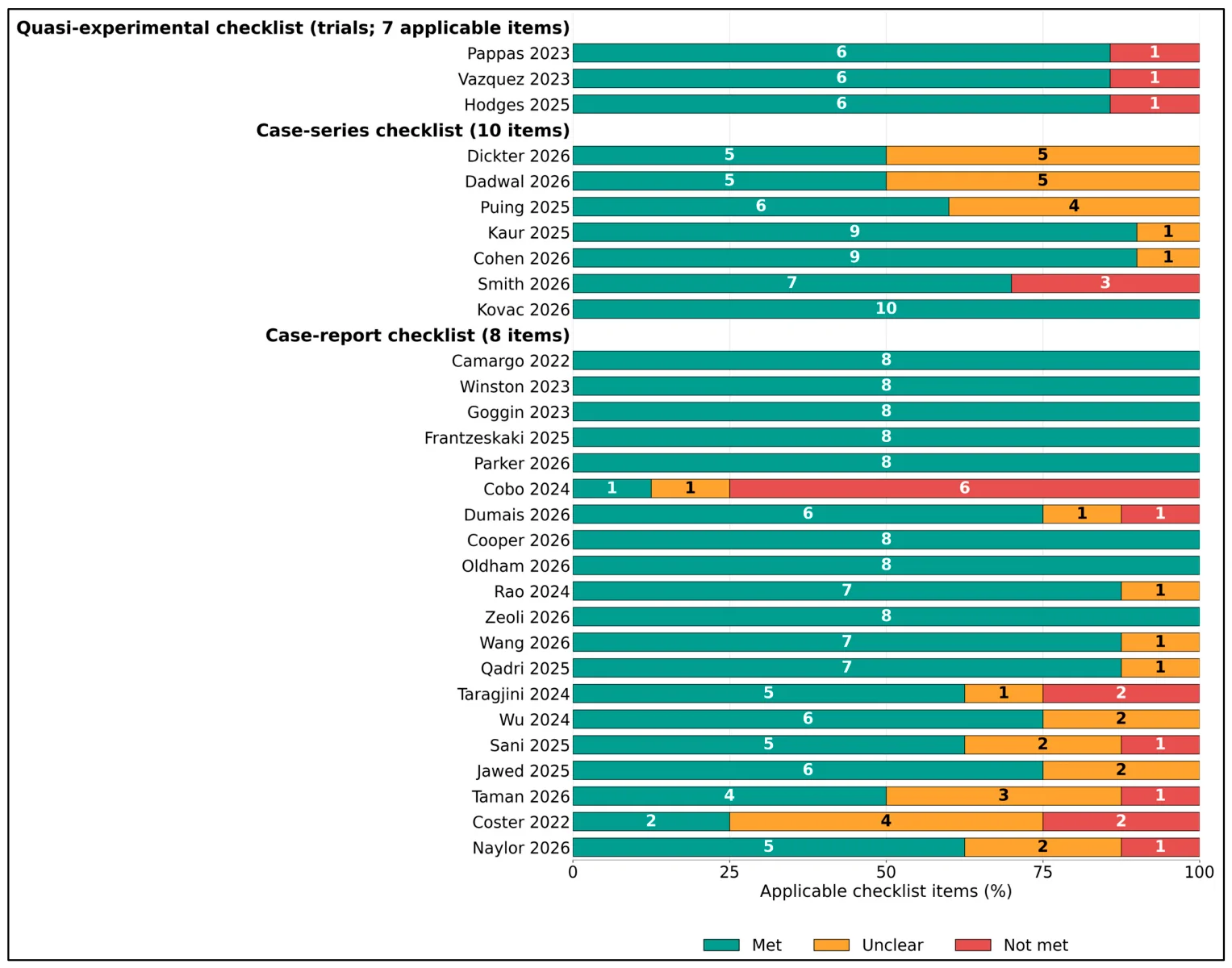
Methodological quality of the 30 included studies appraised with the Joanna Briggs Institute checklists [55,56,57]. Bars show the proportion of applicable checklist items rated as met, unclear or not met for each study, with the number of items in each segment; the two items that do not apply to single-arm trials (comparability of groups and of co-interventions) were excluded from the trial denominators. Studies are grouped by the checklist applied and labelled by first author and year (trials [27,28,29]; expanded-access, outbreak and cohort series [31,32,33,34,52,53,74]; case-level reports [35,36,37,47,49,54,75,76,77,78,79,80,81,82,83,84,85,86,87,88]).

**Table 1:** Characteristics of the 30 included studies (34 reports).

Author	Year	Refs.	Design	Country	n FMGX	n Other	Age (years)	Immune Status
Pappas	2023	[27]	Phase 2, single-arm	USA, IL, ES, BE	21	0	Mean 62.9 (43–78)	Non-IC
Vazquez	2023	[28]	Phase 2, single-arm	South Africa	9	0	Mean 49.8 (21–76)	NR
Hodges (AEGIS)	2025	[29]	Phase 2, single-arm	BE, DE, IL, USA	21	0	Mean 61.9	IC
Dickter (P-1192)	2026	[34]	EAP series, abstract	7 countries	24	0	Median 45.5 (25–75)	IC
Dadwal (P-1351)	2026	[31,73]	EAP series, abstract	7 countries	92	0	Median 50.5 (10–83)	Mixed
Cohen	2026	[33]	EAP series	USA	11	0	Median 35 (19–65)	Mixed
Puing (P4164)	2025	[53]	EAP series, abstract	USA, Belgium	11	0	Median 50 (15–69)	Mixed
Kaur (A182)	2025	[52]	Retrospective case series, abstract	USA	4	17 e	Median 62 (50–76)	IC
Smith (outbreak cohort)	2026	[32,50,51]	Outbreak cohort	USA	7	11 a	Median 31 (23–52) b	Non-IC
Kovac	2026	[74]	Retrospective cohort	USA	2	19 c	Mean 60 (23–83) b	IC
Camargo	2022	[47]	Case report	USA	1	0	40	IC
Winston	2023	[75]	Case report	USA	1	0	26	IC
Goggin	2023	[36]	Case report	USA	1	0	14	IC
Frantzeskaki	2025	[76]	Case report	Greece	1	0	17	Non-IC
Parker	2026	[37]	Case report	USA	1	0	64	IC
Cobo	2024	[77]	Case series (2)	Spain	1	1 d	NR	IC
Dumais	2026	[78]	Case report	USA	1	0	28	IC
Cooper	2026	[35]	Case report	USA	1	0	73	IC
Oldham	2026	[54]	Case series (2)	USA	2	0	15; 10	Non-IC
Rao	2024	[79]	Case report	USA	1	0	63	IC
Zeoli	2026	[80]	Case report	USA	1	0	72	Non-IC
Wang	2026	[81]	Case series (3)	USA	1	2 d	75	IC
Qadri	2025	[82]	Case report, preprint	NR	1	0	27	IC
Taragjini	2024	[83]	Case series (2)	USA	1	1 d	71	IC
Wu	2024	[84]	Case report, abstract	USA	1	0	55	IC
Sani	2025	[85]	Case report, abstract	USA	1	0	47	Non-IC
Jawed	2025	[86]	Case report, abstract	USA	1	0	59	Non-IC
Taman	2026	[87]	Case report, abstract	USA	1	0	NR	IC
Coster	2022	[48,49]	Case report, abstract	USA	1	0	22	IC
Naylor	2026	[88]	Case report, abstract	USA	1	0	52	IC

FMGX, fosmanogepix; EAP, expanded-access programme; IC, immunocompromised (haematological malignancy, transplantation, or primary immunodeficiency); Non-IC, not immunocompromised; NR, not reported. a—Antifungal-treated patients who did not receive fosmanogepix. b—Whole cohort; fosmanogepix subgroup not reported. c—Cohort patients treated with other regimens. d—Patients in the same report not treated with fosmanogepix. e—Other patients with invasive fusariosis identified in the same database (treatment not described). Country codes: IL, Israel; ES, Spain; BE, Belgium; DE, Germany.

**Table 2:** Infection characteristics and fosmanogepix regimens in the included studies.

Study	Refs.	Pathogen	Site	Line	MGX MIC/MEC (µg/mL)	IV Load	Maintenance Dose	Route	Duration	Comb.	Surg.
Pappas 2023	[27]	*Candida* spp.	Bloodstream	1st	0.002–0.03	Yes	600 mg IV/700 mg PO QD	IV→PO (10/21)	≤14 d	No	NA
Vazquez 2023	[28]	*C. auris*	Bloodstream	2nd	0.008–0.015	Yes	600 mg IV QD	IV	Mean 19 d (11–27)	No	NA
Hodges 2025	[29]	Mixed a	Lung 80%	Salvage	0.008–0.03 b	Yes	600 mg IV/800 mg PO QD	IV→PO (NR)	Median 39 d (4–43)	No	NR
Dickter 2026	[34]	*Aspergillus* spp.	Lung, dissem.	Salvage	NR	NR	NR	NR	Median 112 d c; 76 d d	NR	NR
Dadwal 2026	[31,73]	*Fusarium* spp.	Dissem., CNS, bone, skin	Salvage	NR (low)	NR	NR	NR	Median 88.5 d c; 69 d d (100 d c; 102 d d) f	NR	NR
Puing 2025	[53]	Mucorales e	Lung 5; sinus 3; skin 2; liver 1	Salvage (10); cont. (1)	NR (variable)	3/11	800 mg PO QD	PO (8); IV→PO (3)	Median 89 d (9–188)	Yes	NR
Kaur 2025	[52]	*F. solani*	Dissem. (4/4)	Salvage	NR (azole-R)	NR	NR	NR	NR (switch at median 8 d)	No	3/4
Cohen 2026	[33]	*Coccidioides* spp.	CNS 8; dissem. 3	Salvage	NR	2/11	800 mg PO QD	PO (9); IV→PO (2)	Median 208 d (55–1086)	Yes (≥8/11)	NR
Smith 2026	[32,50,51]	*F. solani* complex	CNS	Salvage	≤0.008	NR	NR	NR	NR	Yes	NR
Kovac 2026	[74]	*F. solani*	Dissem.	Salvage	<0.008	NR	NR	NR	NR	Yes	1/2
Camargo 2022	[47]	*A. calidoustus*	Dissem., heart, CNS	Salvage	≤0.008	Yes	600 mg IV QD; 400 mg PO BID	IV→PO	≥191 d	Yes	Yes
Winston 2023	[75]	*F. lactis*	Bloodstream, CNS	Salvage	0.015	Yes	600 mg IV/800 mg PO QD	IV→PO	10 mo	Yes (2 wk)	No
Goggin 2023	[36]	*F. solani* complex	Skin (chronic)	Salvage	≤0.008	No	800 mg QD; 400 mg BID	PO	≈6 mo	No	No
Frantzeskaki 2025	[76]	*Fusarium* sp.	Dissem., CNS	Salvage	NR (low)	No	600 mg IV QD; 400 mg PO BID	IV→PO	≈10 mo	Yes	Yes
Parker 2026	[37]	*F. solani* complex	Skin/soft tissue	Salvage	0.015	No	800 mg QD; 400 mg BID	PO	3 mo (+ re-treatment)	No	No
Cobo 2024	[77]	*L. prolificans*	Bloodstream	NR	NR	NR	NR	NR	NR	NR	NR
Dumais 2026	[78]	*L. prolificans*	Dissem., CNS, gallbladder	Salvage	≤0.008	Yes	600 mg IV/800 mg PO QD	IV→PO	NR	Yes (4 d)	Yes
Cooper 2026	[35]	*L. prolificans*	Dissem., CNS, eye	Salvage	≤0.008	No	800 mg PO QD; 600 mg IV QD	PO→IV	42 d	No	Yes
Oldham 2026	[54]	*Rhizopus* spp.	Rhino-orbital-cerebral	Salvage	2; NR	1/2	800 mg PO QD	PO; IV→PO	6 mo; ≥3 mo	Yes	Yes
Rao 2024	[79]	*S. apiospermum*	Dissem.	Salvage	NR	NR	NR	NR	2 d	Yes	No
Zeoli 2026	[80]	*F. falciforme*	Cornea, anterior chamber	Salvage	≤0.008	Yes	600 mg IV/800 mg PO QD	IV→PO	29 d	Yes (local)	Yes
Wang 2026	[81]	*Fusarium* sp.	Skin, lung	Salvage	NR	NR	NR	IV	4 d	NR	No
Qadri 2025	[82]	*F. solani*	Dissem., CNS, eye	1st (comb.)	NR	NR	NR	IV→PO	≥18 mo	Yes	Yes
Taragjini 2024	[83]	*Fusarium* sp.	Aorta	Cont.	NR	NR	NR	NR	NR	No	Yes
Wu 2024	[84]	*F. solani*	Lung/airway	Salvage	NR	NR	NR	PO	4 mo	No	No
Sani 2025	[85]	*L. prolificans*	Bone	Salvage	NR	NR	NR	IV→PO	≥34 d	NR	Yes
Jawed 2025	[86]	*C. auris*	Bloodstream	Salvage	NR	NR	NR (QD)	NR	15 doses	NR	NR
Taman 2026	[87]	*C. bantiana*	CNS	Salvage	NR	NR	NR	NR	NR	Yes (local)	Yes
Coster 2022	[48,49]	*L. prolificans*	Lung/airway	Salvage	NR	NR	NR	NR	NR	Yes	Yes
Naylor 2026	[88]	*C. auris* + *M. circinelloides*	Bloodstream, abdomen	Salvage	NR (susceptible)	NR	NR	NR	≥18 d	NR	NR

MGX, manogepix; MIC, minimum inhibitory concentration; MEC, minimum effective concentration; IV, intravenous; PO, oral; QD, once daily; BID, twice daily; d, days; mo, months; dissem., disseminated; CNS, central nervous system; Line, line of fosmanogepix therapy (1st, first-line; 2nd, after echinocandin; salvage, after failure of or intolerance to standard agents; continuation, after completion of standard therapy); IV load, intravenous loading dose of 1000 mg twice on day 1 (Yes/No/NR; fraction where mixed); Comb., concomitant systemic antifungal during fosmanogepix (local, intraocular or intracavitary antifungal only); Surg., surgery or source control; cont., continuation therapy; NA, not applicable; NR, not reported. a—*Aspergillus* spp., *Fusarium* spp., *Lomentospora prolificans*, Mucorales; pathogen unidentified in 12 participants. b—Except *Mucor circinelloides* (4 µg/mL). c—Haematological subgroup. d—Non-haematological subgroup. e—*Rhizopus* 3, *Mucor* 3, Rhizomucor 1, unspecified Mucorales 4; 3 mixed infections with *Aspergillus fumigatus*, *Fusarium* or Curvularia. f—Values in parentheses from the earlier ESCMID 2025 data cut (81 patients).

**Table 3:** Treatment outcomes, safety and methodological quality of the included studies.

Study	Refs.	n	Response (n/N or Status)	Mortality (n/N; Time)	Follow-Up	Relapse	AE Due to FMGX	GI AE	Stop for AE	JBI Items
Pappas 2023	[27]	21	16/20 (80%) a	3/20; day 30	4 wk post-EOT	2/20	1/21	NR b	0/21	6/7
Vazquez 2023	[28]	9	8/9 (89%) a	1/9; day 30	4 wk post-EOST	1/9	0/9	NR b	0/9	6/7
Hodges 2025	[29]	21	8/20 (40%) c	5/20; day 42 (8/20; day 84)	Day 84	NR	15/21	11/21 nausea	3/21 d	6/7
Dickter 2026	[34]	24	13/17; 4/7 e	4/17; 3/7; 6 wk	≤6 wk	NR	NR	NR	1/24	5/10
Dadwal 2026	[31,73]	92	38/50; 26/32 e	16/50 f; 0/32; 6 wk	≤6 wk	NR	NR	Most common	3/92	5/10
Puing 2025	[53]	11	7/11 (64%) l	2/11; day 14 and 156	To end of FMGX	NR	2/11	2/11	2/11	6/10
Kaur 2025	[52]	4	4/4 (100%) m	0/4; 12 wk	12 wk	NR	1/4	1/4	1/4	9/10
Cohen 2026	[33]	11	8/11 (73%) g	2/11; to cut-off	To cut-off	NR	6/11	6/11	NR	9/10
Smith 2026	[32,50,51]	7	NR (survival only)	1/7 vs. 8/11 h	NR	NR	NR	NR	NR	7/10
Kovac 2026	[74]	2	PR 1; failure 1	2/2; day 963 and 41	To death	NR	NR	NR	NR	10/10
Camargo 2022	[47]	1	Favourable	0/1; day 324	191 d on FMGX	0/1	0/1	No	No	8/8
Winston 2023	[75]	1	Favourable	0/1	16 mo off FMGX	0/1	0/1	No	No	8/8
Goggin 2023	[36]	1	Favourable	0/1	3 mo off FMGX	0/1	1/1	Yes	No	8/8
Frantzeskaki 2025	[76]	1	Favourable	0/1	Day 533	0/1	1/1	Yes	No	8/8
Parker 2026	[37]	1	Favourable	0/1	9 mo	Possible	1/1	Yes	No	8/8
Cobo 2024	[77]	1	Favourable	0/1	NR	NR	NR	NR	NR	1/8
Dumais 2026	[78]	1	Favourable	1/1 i; day 100	≈90 d	0/1	NR	NR	NR	6/8
Cooper 2026	[35]	1	Failure	1/1; day 73	To death	NA	Possible	Yes	Held 6 d	8/8
Oldham 2026	[54]	2	Favourable (2)	0/2	>2 y; 1 y	0/2	1/2	1/2	No	8/8
Rao 2024	[79]	1	Not assessable	1/1; day 9	To death	NA	1/1	Yes	Hospice	7/8
Zeoli 2026	[80]	1	Favourable	0/1	2 y	0/1	0/1 j	No	No	8/8
Wang 2026	[81]	1	Partial	1/1 k; NR	2 wk	NA	NR	Yes o	NR	7/8
Qadri 2025	[82]	1	Favourable	0/1	18 mo	Progression p	0/1	No	No	7/8
Taragjini 2024	[83]	1	Indeterminate	0/1; 2 mo	2 mo	NR	NR	NR	NR	5/8
Wu 2024	[84]	1	Favourable	0/1	4 mo	0/1	0/1	No	No	6/8
Sani 2025	[85]	1	Favourable	0/1	34 d	NR	NR	NR	NR	5/8
Jawed 2025	[86]	1	Favourable	0/1	NR	NR	0/1	No	No	6/8
Taman 2026	[87]	1	Favourable	0/1	2 y	0/1	0/1	No	No	4/8
Coster 2022	[48,49]	1	Indeterminate	0/1	1 y	NR	NR	NR	NR	2/8
Naylor 2026	[88]	1	Favourable	0/1	NR	NR	NR	NR	NR	5/8

AE, adverse event; GI, gastrointestinal (nausea, vomiting or diarrhoea); EOT, end of treatment; EOST, end of study treatment; PR, partial response; NA, not applicable; NR, not reported. For case-level reports, Response is the reviewer classification of the last reported status (rules in Section 3.4). JBI items met: quasi-experimental checklist (7 applicable items) for trials, case-series checklist (10 items) for series and cohorts, case-report checklist (8 items) for case-level reports. a—Data-review-committee success at end of study treatment (modified intention-to-treat). b—Diarrhoea and vomiting, each in 3/21 treatment-emergent events in the candidaemia trial, attribution not stated; not reported separately for *C. auris*. c—Data-review-committee global response (complete or partial). d—Discontinuation for drug-related events; 7/21 stopped for any adverse event. e—Haematological; non-haematological subgroup. f—Died (9) or entered hospice (7). g—Author-adjudicated success. h—Fosmanogepix recipients versus antifungal-treated non-recipients. i—Death from relapsed leukaemia after resolution of infection. j—Flank pain attributed to a benign liver cyst. k—Death from bacterial sepsis with persistent pulmonary fusariosis. l—Physician-assessed favourable response (clinical and/or radiological improvement). m—Mycological and radiological response in all four patients. o—Diarrhoea and constipation, not attributed to fosmanogepix. p—New lesions during therapy without discontinuation.

**Table 4:** Reported outcomes stratified by study design and by concomitant systemic antifungal therapy.

Evidence Stratum	Study [Refs.]	Patient-Episodes	Concomitant Systemic Antifungal	Outcome Measure	Favourable Outcome, n/N (%)	Deaths, n/N (Time)
Phase 2 trials, candidaemia	Pappas 2023; Vazquez 2023 [27,28]	30	None (monotherapy by protocol)	DRC-adjudicated success at EOST	16/20 (80); 8/9 (89)	3/20; 1/9 (day 30)
Phase 2 trial, refractory invasive mould disease	Hodges 2025 [29]	21	None (monotherapy by protocol)	DRC-adjudicated global response, day 42	8/20 (40)	5/20 (day 42); 8/20 (day 84)
EAP series, aspergillosis (abstract)	Dickter 2026 [34]	24	NR	Physician-assessed favourable response	13/17 (76) a; 4/7 (57) b	4/17; 3/7 (6 wk)
EAP series, fusariosis (abstract)	Dadwal 2026 [31,73]	92	NR	Physician-assessed favourable response	38/50 (76) a; 26/32 (81) b	16/50 c; 0/32 (6 wk)
EAP series, mucormycosis (abstract)	Puing 2025 [53]	11	Yes, 10/11 (L-AmB, isavuconazole or posaconazole)	Physician-assessed favourable response	7/11 (64)	2/11 (days 14 and 156)
EAP series, coccidioidomycosis	Cohen 2026 [33]	11	Yes, ≥8/11	Author-adjudicated success	8/11 (73)	2/11 (to cut-off)
Single-centre series, fusariosis after transplantation	Kaur 2025 [52]	4	None (switched to monotherapy)	Mycological and radiological response	4/4 (100)	0/4 (12 wk)
Outbreak cohort, *Fusarium* meningitis	Smith 2026 [32,50,51]	7	Yes	Survival only	NA	1/7 (vs. 8/11 non-recipients)
Retrospective cohort, fusariosis	Kovac 2026 [74]	2	Yes	Reviewer classification of last status	0/2 (1 partial, 1 failure)	2/2 (days 963 and 41)
Case-level, concomitant systemic antifungal	[47,48,49,54,75,76,78,79,82]	9	Yes (L-AmB, isavuconazole, voriconazole or other)	Reviewer classification of last status	7/9 (78) d	2/9 e
Case-level, fosmanogepix as sole systemic antifungal	[35,36,37,80,83,84,87]	7	No (local antifungal only in 2)	Reviewer classification of last status	5/7 (71) f	1/7 g
Case-level, concomitant therapy not reported	[77,81,85,86,88]	5	NR	Reviewer classification of last status	4/5 (80) h	1/5 i

DRC, data review committee; EOST, end of study treatment; EAP, expanded-access programme; L-AmB, liposomal amphotericin B; NA, not applicable; NR, not reported; wk, weeks. Strata are presented separately and were not combined into any aggregate estimate. a—Haematological subgroup. b—Non-haematological subgroup. c—Died (9) or entered hospice (7). d—One not assessable (two days of therapy) and one indeterminate. e—One death from relapsed leukaemia after resolution of infection and one after election of hospice on day 2 of therapy. f—One failure (progressive *L. prolificans* meningitis) and one indeterminate. g—Progressive *L. prolificans* meningitis. h—One partial response. i—Death from bacterial sepsis with persistent pulmonary fusariosis.

**Table 5:** GRADE-informed assessment of the certainty of evidence for each pathogen-indication pair.

Indication	Evidence Base (Design; Patient-Episodes) [Refs.]	Key Outcome (Source-Reported)	Main Reasons for Downgrading	Consistency Across Sources	GRADE-Informed Certainty	Implication for Practice
Candidaemia (non-*C. auris*)	1 phase 2 single-arm trial; 21 [27]	DRC success 16/20 (80%); Day-30 mortality 3/20	Very serious imprecision; indirectness (non-neutropenic patients, ≤14 days, deep-seated infection excluded); no comparator	Single source	Low	Supports the ongoing phase 3 comparison; not a basis for routine use
*C. auris* candidaemia	1 phase 2 trial (9) + 2 case-level abstracts (2); 11 [28,86,88]	DRC success 8/9 (89%); 2/2 favourable in abstracts	Very serious imprecision; early termination; abstract-level case data	Consistent direction, all favourable	Very low	Option where echinocandins fail or cannot be used, within trials or expanded access
Refractory invasive mould disease (mixed or unidentified moulds)	1 phase 2 single-arm trial; 21 [29]	Global response 8/20 (40%); Day-42 mortality 5/20 (25%)	Very serious imprecision; indirectness (historical benchmark; pathogen unidentified in 12); 33% discontinuation	Single source; conflicts with expanded-access responses	Low	Survival and disease control may diverge; salvage use only
Invasive aspergillosis	1 EAP abstract (24) + 1 case report (1); 25 [34,47]	Physician-assessed favourable 13/17 and 4/7	Serious risk of bias (unmonitored, physician-assessed, no concomitant-therapy data); imprecision; suspected reporting bias	Two sources, both favourable	Very low	Salvage use after azole and amphotericin B failure; no basis for earlier use
Fusariosis	1 EAP abstract with 2 data cuts (92), 1 single-centre series (4), 1 outbreak cohort (7), 1 cohort (2), 9 case reports (9); 114 [31,32,36,37,52,73,74,75,76,80,81,82,83,84]	Physician-assessed favourable 38/50 and 26/32; 4/4 in transplant series; 6/7 survival in outbreak cohort	Serious risk of bias; unresolved overlap; concomitant therapy unreported in the largest source; suspected reporting bias	Consistent direction across five source types	Very low	One of the more extensively documented investigational options for refractory disease; highest priority for a comparative trial
Mucormycosis	1 EAP abstract (11) + 1 paediatric case series (2); 13 [53,54]	Physician-assessed favourable 7/11 (64%); 2/2 favourable in children	Serious risk of bias; imprecision; indirectness (10/11 on continued amphotericin B or azole; variable manogepix MIC)	Two sources, both favourable	Very low	Adjunctive or expanded-access use only; contribution of fosmanogepix not separable
Coccidioidomycosis	1 retrospective EAP series; 11 [33]	Author-adjudicated success 8/11 (73%), including 6/8 with meningitis	Risk of bias (retrospective author adjudication; ≥8/11 on concomitant therapy); imprecision	Single source	Very low	Salvage option for refractory meningitis pending prospective data
*Scedosporium* or *Lomentospora* infection	6 case reports or abstracts; 6 [35,48,49,77,78,79,85]	3/6 favourable; 1 failure; 1 indeterminate; 1 not assessable	Case-level evidence only; serious imprecision; suspected reporting bias; documented failure despite low MEC	Inconsistent (one fatal progression)	Very low	Use with caution; susceptibility may not predict response
Tolerability of prolonged therapy (all indications)	3 trials (51) + 4 series with median exposure of 69 to 208 days (138) [27,28,29,31,33,34,53]	Drug-related discontinuation 3/51 in trials; GI events predominant; no organ toxicity reported	Incomplete adverse-event reporting outside trials; imprecision for uncommon events	Consistent across all source types	Low	GI intolerance expected; hepatic, renal and cardiac safety not excluded for rare events

DRC, data review committee; EAP, expanded-access programme; GI, gastrointestinal; MEC, minimum effective concentration; MIC, minimum inhibitory concentration. Each body of evidence started at low certainty, the GRADE starting point for non-randomised designs, and was downgraded one level (to very low) when at least one serious concern beyond imprecision applied; no body of evidence met any upgrading criterion. “Consistency across sources” is descriptive and did not raise any rating, because the sources within each indication share the same biases.

## Data Availability

All data analysed in this review were extracted from published reports cited in the reference list. The extraction records are available from the corresponding author on request.

## Abbreviations

The following abbreviations are used in this manuscript:

AE	adverse event
AmB	amphotericin B
AML	acute myeloid leukaemia
BID	twice daily
CI	confidence interval
CNS	central nervous system
DRC	data review committee
EAP	expanded-access programme
EOST	end of study treatment
EOT	end of treatment
FMGX	fosmanogepix
GI	gastrointestinal
GRADE	Grading of Recommendations Assessment, Development and Evaluation
IC	immunocompromised
IFD	invasive fungal disease
IV	intravenous
JBI	Joanna Briggs Institute
L-AmB	liposomal amphotericin B
MEC	minimum effective concentration
MGX	manogepix
MIC	minimum inhibitory concentration
mITT	modified intention-to-treat
NR	not reported
PO	oral
PR	partial response
QD	once daily
SOT	solid-organ transplantation

## Appendix A

Scenario analyses of patient duplication (Table A1 and Table A2) and reporting bias (Table A3), as described in Section 2.5. Source-reported figures are retained in the main text; the scenarios bound the plausible effect of duplication and selective reporting on the counts and proportions that could not be verified at patient level.

**Table A1:** Estimated number of unique patients by pathogen group under duplication scenarios.

Pathogen Group	Source-Reported Patient-Episodes (Trial/Non-Trial)	Resolved Overlap a	Maximal Overlap b	10% Duplication c	20% Duplication c	30% Duplication c
*Fusarium* spp.	114 (0/114)	106	92	103	91	80
*Candida* spp.	32 (30/2)	32	32	32	32	31
*Aspergillus* spp.	25 (0/25)	25	24	23	20	18
Mucorales	13 (0/13)	12	10	12	10	9
*Coccidioides* spp.	11 (0/11)	11	11	10	9	8
*Scedosporium* or *Lomentospora* spp.	6 (0/6)	6	6	5	5	4
*Cladophialophora bantiana*	1 (0/1)	1	1	1	1	1
Mixed or unidentified moulds (mould trial)	21 (21/0)	21	21	21	21	21
All pathogens	223 (51/172)	214	197	206	189	171

a—Removal of the two patients enrolled in both a phase 2 trial and a series [52,53] (removed from the series count) and of the seven outbreak-cohort recipients [32] assumed to be among the eight United States outbreak patients in the fusariosis abstract [31]. b—Scenario a plus the assumption that every non-trial patient-episode with *Fusarium*, *Aspergillus* or Mucorales infection reported outside the programme abstracts (22, 1 and 2 patient-episodes, respectively) is included in those abstracts. c—Uniform duplication rate applied to the non-trial patient-episodes and rounded to the nearest integer; trial participants are treated as unique. Per-pathogen values are rounded independently and need not sum to the overall total.

**Table A2:** Bounding ranges for case-level outcomes (21 individually described patients) under duplication scenarios.

Scenario	Patient-Episodes Retained	Favourable Outcome, Best Case n/N (%)	Favourable Outcome, Worst Case n/N (%)	Alive at Last Report, Best Case n/N (%)	Alive at Last Report, Worst Case n/N (%)
Source-reported	21	16/21 (76)	16/21 (76)	17/21 (81)	17/21 (81)
Resolved overlap a	21	16/21 (76)	16/21 (76)	17/21 (81)	17/21 (81)
Maximal overlap b	9	6/9 (67)	6/9 (67)	6/9 (67)	6/9 (67)
10% duplication (2 removed)	19	16/19 (84)	14/19 (74)	17/19 (89)	15/19 (79)
20% duplication (4 removed)	17	16/17 (94)	12/17 (71)	17/17 (100)	13/17 (76)
30% duplication (6 removed)	15	15/15 (100)	10/15 (67)	15/15 (100)	11/15 (73)

Best case assumes that the removed duplicates were all unfavourable (or had died); worst case assumes that they were all favourable (or alive). Unfavourable comprises partial (1), indeterminate (2), failure (1) and not assessable (1). a—No case-level patient was involved in definite or probable overlap. b—Removal of the 12 case-level patients with *Fusarium*, *Aspergillus*, or Mucorales infection [36,37,47,54,75,76,80,81,82,83,84], whose outcomes are known, so best and worst cases coincide.

**Table A3:** Reporting-bias scenario: unpublished treatment failures implied by the published case-level literature.

Assumed True Favourable Proportion Among Individually Treated Patients	Basis for the Assumption	Implied Total Patients for 16 Published Favourable Outcomes	Implied Unfavourable Outcomes	Unpublished Failures Implied (5 Unfavourable Published)
76%	Physician-assessed favourable response in expanded-access fusariosis, haematological subgroup [31]	21	5	0
64%	Physician-assessed favourable response in expanded-access mucormycosis [53]	25	9	4
50%	Illustrative midpoint	32	16	11
40%	Adjudicated global response in the phase 2 mould trial [29]	40	24	19

Implied total = 16 divided by the assumed proportion, rounded to the nearest integer; implied unfavourable = implied total minus 16; unpublished failures = implied unfavourable minus the five published unfavourable outcomes (one failure, one partial response, two indeterminate and one not assessable). The calculation assumes that the 16 published favourable outcomes are the complete set of favourable outcomes, which makes the implied number of unpublished failures conservative.
